# Metastatic organ distribution characteristics and prognosis in patients with non-small cell lung cancer

**DOI:** 10.3389/fmed.2026.1917484

**Published:** 2026-08-04

**Authors:** Jianlei Zhu, Wenyan Pan, Yanyang Wang

**Affiliations:** 1 Department of Radiotherapy, Ningxia Medical University General Hospital, Yinchuan, Ningxia, China; 2 Department of Medical Oncology, Yinchuan First People’s Hospital, Yinchuan, Ningxia, China

**Keywords:** metastasis, non-small cell lung cancer, organ distribution, overall survival, prognosis

## Abstract

**Background:**

Non-small cell lung cancer (NSCLC) is often diagnosed at advanced stages, with distant metastasis being the leading cause of death. However, the distribution patterns of metastatic organs and their prognostic impact remain unclear. This study aimed to characterize the distribution of metastatic organs and evaluate their prognostic significance in patients with metastatic NSCLC.

**Methods:**

This retrospective study included 305 patients with metastatic NSCLC diagnosed at our institution between 1 January 2022 and 1 January 2026. Patients were categorized into the single-organ metastasis (SOM) group (*n* = 159) and the multiple-organ metastasis (MOM) group (*n* = 146). Metastatic sites assessed included the brain, bone, liver, lung, and adrenal glands. Overall survival (OS) was compared between the groups using the Kaplan–Meier analysis and log-rank tests. Univariate and multivariate Cox regression models were used to identify factors associated with OS.

**Results:**

The most common metastatic site was the bone in both the SOM (40.9%) and MOM (82.2%) groups. Patients in the MOM group exhibited significantly worse OS than those in the SOM group (median OS: 3.22 vs. 8.22 months, log-rank test *p* < 0.05). A multivariate Cox regression analysis identified the MOM pattern (hazard ratio [HR] = 2.60), older age (HR = 1.03), N2 stage (HR = 1.71), and N3 stage (HR = 1.86) as independent risk factors for poorer OS, while chemotherapy (HR = 0.63) and immunotherapy (HR = 0.54) were protective factors (all *p* < 0.05). Among patients with SOM, the median OS was 9.09, 7.06, 4.99, 21.06, and 5.09 months for the bone, brain, liver, lung, and adrenal gland metastases, respectively. Patients with adrenal gland metastasis had significantly worse OS than those with bone or lung metastases (*p* < 0.05).

**Conclusion:**

Multiple-organ metastasis independently predicts poorer prognosis. In patients with SOM, preliminary evidence suggests that adrenal gland involvement may be associated with worse survival than bone or lung metastases, whereas no significant differences were observed among the other sites. These findings highlight the importance of assessing both the extent and distribution of metastatic involvement for risk stratification.

## 1 Introduction

Lung cancer is the most frequently diagnosed malignancy and is the leading cause of cancer-related death globally (1). According to the Global Cancer Statistics 2022, an estimated 2.48 million new cases and 1.82 million deaths were attributed to lung cancer, representing the highest mortality among all cancers (1). Non-small cell lung cancer (NSCLC) constitutes approximately 85% of all lung cancer cases, including adenocarcinoma, squamous cell carcinoma, and large cell carcinoma (2). Despite substantial progress in targeted therapies and immune checkpoint inhibitors, the majority of patients with NSCLC are diagnosed at an advanced stage (3). In addition, distant metastasis is the predominant cause of treatment failure and disease-related deaths (4).

The patterns of metastatic spread in NSCLC vary considerably across patients. Common metastatic sites include the brain, bone, liver, lung, and adrenal glands (5). Each site presents distinct clinical challenges and may exert a different impact on patient prognosis. For instance, brain metastases frequently lead to neurological deficits and are associated with poor survival outcomes, with median overall survival (OS) rarely exceeding 12 months without aggressive local therapy (6). Bone metastases, while often not immediately life-threatening, can cause severe pain, pathological fractures, and spinal cord compression, significantly impairing patients’ quality of life (7). Liver metastases have been increasingly recognized as an independent negative prognostic factor, possibly due to altered drug metabolism and immune tolerance mechanisms (8).

Previous studies have typically evaluated individual metastatic sites in mixed cohorts or simply distinguished between oligometastatic and polymetastatic disease. However, a systematic comparison of survival outcomes across the five major metastatic sites and the corresponding prognostic hierarchy in patients with truly single-organ metastasis has not been established, despite its potential value for guiding site-specific surveillance and local therapy decisions. Existing evidence is conflicting: A large Surveillance, Epidemiology, and End Results (SEER) analysis reported better overall survival for isolated adrenal metastases than for liver or brain involvement (9), whereas another study identified multiple-organ involvement, rather than any single site, as the strongest predictor of early death (10). These discrepancies likely arise from reliance on population-based registries with limited clinical detail and from the common practice of dichotomizing patients without accounting for organ-specific effects. Therefore, a comprehensive analysis of metastatic distribution in a well-characterized contemporary cohort is warranted to clarify the independent prognostic impact of both the number and location of involved metastatic organs, thereby refining risk stratification and informing personalized management.

In this retrospective study, we analyzed clinical and follow-up data from patients with metastatic NSCLC diagnosed at our institution between 1 January 2022 and 1 January 2026. This study aimed to describe the frequency and distribution patterns of metastatic involvement in the brain, bone, liver, lung, and adrenal glands and to investigate the association between different metastatic sites, as well as single-organ and multiple-organ involvement, with OS in patients with metastatic NSCLC. Our findings may help identify high-risk patient subgroups and inform more effective surveillance and treatment strategies for advanced NSCLC.

## 2 Methods

### 2.1 Study design and patient population

This retrospective study included patients with metastatic NSCLC diagnosed at our institution between 1 January 2022 and 1 January 2026. The inclusion criteria were as follows: (1) histopathologically confirmed NSCLC; (2) evidence of distant metastasis at initial diagnosis or during follow-up; and (3) complete clinical and follow-up data. The exclusion criteria were as follows: (1) concurrent primary malignancies; (2) incomplete staging workup; and (3) missing survival outcome data. Finally, a total of 305 patients met the inclusion criteria.

The study protocol was approved by the Ethics Committee of Yinchuan First People’s Hospital (No. zy-2026-131). Informed consent was waived due to the retrospective nature of the study and the use of anonymized clinical data. This study was conducted in accordance with the Strengthening the Reporting of Observational Studies in Epidemiology (STROBE) guidelines.

### 2.2 Data collection

Clinical and pathological data were extracted from electronic medical records. The following variables were collected: age at diagnosis, sex, smoking status (ever smoker vs. never smoker), pathological type (adenocarcinoma, squamous cell carcinoma, or other), T stage (T1–T4) and N stage (N0–N3) based on the 8th edition of the AJCC TNM classification (11), and treatment modalities including chemotherapy, radiotherapy, immunotherapy, and targeted therapy. In addition, hypertension and diabetes mellitus were extracted from the medical history records. Information on local treatment directed at metastatic lesions, including surgery, stereotactic radiotherapy, or ablation, defined as any local therapy administered to metastatic sites, was also collected as a binary variable (yes/no) based on available procedure documentation.

Metastatic organ involvement was determined by imaging studies, including contrast-enhanced computed tomography, magnetic resonance imaging, or positron emission tomography (PET) (12). The metastatic sites assessed included the brain, bone, liver, contralateral or ipsilateral lung, and adrenal glands. Patients were categorized into two groups based on the number of involved metastatic organs (13): a single-organ metastasis (SOM) group, defined as metastasis to only one of the five specified organs, which comprised patients with either a single metastatic lesion or multiple lesions confined to that organ, and a multiple-organ metastasis (MOM) group, defined as metastasis to two or more of these organs.

### 2.3 Outcomes

OS was defined as the time from the date of metastatic disease diagnosis to death from any cause or to the date of last follow-up (14). For patients with metastasis at initial diagnosis, this was the date of initial diagnosis; for patients who developed metastasis during follow-up, this was the date of first radiographic confirmation of distant metastasis. Patients who were alive at the end of the study or were lost to follow-up were censored. Patients who were lost to clinical follow-up for more than 6 months were censored at the date of last contact.

### 2.4 Statistical analysis

Statistical analyses were conducted using SPSS version 26.0. Normality of continuous variables was assessed using the Shapiro–Wilk test. Normally distributed data were presented as mean ± standard deviation (x̄ ± s) and were compared using the independent samples t-test; otherwise, the Mann–Whitney U test was used. Categorical variables were presented as frequencies and percentages and were compared using the chi-square (*χ*^2^) test. Univariate and multivariate Cox proportional hazards regression models were used to identify factors associated with OS. The proportional hazards assumption was verified using the Schoenfeld residual tests. Survival curves were estimated using the Kaplan–Meier method and compared using the log-rank test. Within the SOM group, pairwise comparisons of OS between specific metastatic sites were performed using the log-rank test. All statistical tests were two-sided, and a *p-*value of < 0.05 was considered statistically significant.

## 3 Results

### 3.1 Patient characteristics

A total of 305 patients with metastatic NSCLC were included, with 159 patients in the SOM group and 146 patients in the MOM group. Among the SOM group, 41 patients (25.8%) had a single metastatic lesion within the involved organ, while 118 patients (74.2%) had multiple lesions confined to a single organ. The baseline characteristics are summarized in Table 1. The mean age was 61.8 ± 10.2 years in the SOM group and 63.0 ± 8.7 years in the MOM group, with no significant difference (*p* > 0.05). Significant differences were observed in smoking status and N stage between the two groups (all *p* < 0.05). However, no significant differences were found for sex, pathological type, T stage, chemotherapy, radiotherapy, immunotherapy, targeted therapy, hypertension, diabetes mellitus, or local treatment (all *p* > 0.05).

**Table 1:** Characteristics in the SOM and MOM groups (*n* = 305).

Characteristics	SOM (*n* = 159)	MOM (*n* = 146)	*χ*^2^/*t*	*p*
Age (years), *x̄* ± *s*	61.8 ± 10.2	63.0 ± 8.7	−1.031	0.304
Sex, *n* (%)			2.144	0.143
Female	63 (39.6)	70 (47.9)		
Male	96 (60.4)	76 (52.1)		
Smoking status, *n* (%)			4.583	**0.032**
No	102 (64.2)	76 (52.1)		
Yes	57 (35.8)	70 (47.9)		
Pathological types, *n* (%)			0.698	0.705
Adenocarcinoma	95 (59.7)	91 (62.3)		
Squamous	44 (27.7)	41 (28.1)		
Others	20 (12.6)	14 (9.6)		
T stage, *n* (%)			5.714	0.126
T_1_	38 (23.9)	30 (20.5)		
T_2_	40 (25.2)	55 (37.7)		
T_3_	48 (30.2)	38 (26.0)		
T_4_	33 (20.8)	23 (15.8)		
N stage, *n* (%)			7.914	**0.048**
N_0_	29 (18.2)	22 (15.4)		
N_1_	28 (17.6)	17 (11.6)		
N_2_	40 (25.2)	27 (18.5)		
N_3_	62 (39.0)	80 (54.8)		
Chemotherapy, *n* (%)			0.538	0.463
No	44 (27.7)	46 (31.5)		
Yes	115 (72.3)	100 (68.5)		
Radiotherapy, *n* (%)			1.018	0.313
No	107 (67.3)	106 (72.6)		
Yes	52 (32.7)	40 (27.4)		
Immunotherapy, *n* (%)			0.413	0.520
No	134 (84.3)	119 (81.5)		
Yes	25 (15.7)	27 (18.5)		
Targeted therapy, *n* (%)			0.166	0.684
No	58 (36.5)	50 (34.2)		
Yes	101 (63.5)	96 (65.8)		
Hypertension, *n* (%)			1.115	0.291
No	103 (64.8)	86 (58.9)		
Yes	56 (35.2)	60 (41.1)		
Diabetes mellitus, *n* (%)			0.895	0.344
No	130 (81.8)	113 (77.4)		
Yes	29 (18.2)	33 (22.6)		
Local treatment, *n* (%)			0.674	0.411
No	133 (83.6)	127 (87.0)		
Yes	26 (16.4)	19 (13.0)		

### 3.2 Prognostic factors for OS

Overall, 231 patients (75.7%) had died, including 99 (62.3%) in the SOM group and 132 (90.4%) in the MOM group. Cox regression analyses were performed to identify prognostic factors for OS (Table 2). The Schoenfeld residual tests confirmed the proportional hazards assumption (global test *p* > 0.05). In the univariate analysis (Model 1), older age (HR = 1.03, 95% CI: 1.01–1.04, *p* < 0.05), N2 stage (HR = 1.65, 95% CI: 1.06–2.56, *p* < 0.05), N3 stage (HR = 1.81, 95% CI: 1.22–2.67, *p* < 0.05), and the MOM pattern (HR = 2.53, 95% CI: 1.93–3.32, *p* < 0.05) were associated with poorer OS. Conversely, chemotherapy (HR = 0.71, 95% CI: 0.54–0.93, *p* < 0.05) and immunotherapy (HR = 0.69, 95% CI: 0.48–0.99, *p* < 0.05) were associated with improved OS. Sex, smoking status, pathological type, T stage, radiotherapy, targeted therapy, hypertension, diabetes mellitus, and local treatment were not significant in the univariate analysis (all *p* > 0.05).

**Table 2:** Cox regression analysis for OS.

Variable	Model 1		Model 2	
	HR (95% CI)	*p*	HR (95% CI)	*p*
Age (years)	1.03 (1.01–1.04)	**< 0.001**	1.03 (1.01–1.04)	**< 0.001**
Sex				
Female	1 (reference)		1 (reference)	
Male	1.22 (0.94–1.59)	0.140	1.29 (0.97–1.71)	0.078
Smoking status				
No	1 (reference)		1 (reference)	
Yes	1.16 (0.90–1.51)	0.253	1.19 (0.90–1.57)	0.231
Pathological types				
Adenocarcinoma	1 (reference)		1 (reference)	
Squamous	1.11 (0.83–1.49)	0.468	1.20 (0.93–1.54)	0.181
Others	0.76 (0.48–1.21)	0.246	1.01 (0.62–1.62)	0.981
T stage				
T_1_	1 (reference)		1 (reference)	
T_2_	1.25 (0.87–1.79)	0.235	1.19 (0.82–1.74)	0.357
T_3_	1.16 (0.80–1.68)	0.439	1.37 (0.92–2.04)	0.123
T_4_	1.34 (0.89–2.02)	0.165	1.70 (0.98–2.65)	0.068
N stage				
N_0_	1 (reference)		1 (reference)	
N_1_	1.32 (0.81–2.16)	0.268	1.60 (0.97–2.65)	0.068
N_2_	1.65 (1.06–2.56)	**0.027**	1.71 (1.17–2.89)	**0.003**
N_3_	1.81 (1.22–2.67)	**0.003**	1.86 (1.22–2.84)	**0.004**
Chemotherapy				
No	1 (reference)		1 (reference)	
Yes	0.71 (0.54–0.93)	**0.014**	0.63 (0.47–0.85)	**0.002**
Radiotherapy				
No	1 (reference)		1 (reference)	
Yes	0.79 (0.59–1.05)	0.109	0.74 (0.55–1.01)	0.058
Immunotherapy				
No	1 (reference)		1 (reference)	
Yes	0.69 (0.48–0.99)	**0.042**	0.54 (0.37–0.79)	**0.001**
Targeted therapy				
No	1 (reference)		1 (reference)	
Yes	0.82 (0.63–1.07)	0.144	0.77 (0.58–1.02)	0.069
Hypertension				
No	1 (reference)		1 (reference)	
Yes	1.14 (0.87–1.48)	0.337	1.16 (0.88–1.52)	0.297
Diabetes mellitus				
No	1 (reference)		1 (reference)	
Yes	0.91 (0.66–1.25)	0.550	0.92 (0.66–1.30)	0.642
Local treatment				
No	1 (Reference)		1 (reference)	
Yes	0.70 (0.47–1.04)	0.076	0.76 (0.50–1.16)	0.208
Transfer pattern				
SOM	1 (reference)		1 (reference)	
MOM	2.53 (1.93–3.32)	**< 0.001**	2.60 (1.96–3.46)	**< 0.001**

Model 1: univariate Cox regression for each variable. Model 2: multivariate Cox regression with full adjustment for all covariates. HR = hazard ratio; CI = confidence interval.

In the multivariate analysis (Model 2), after adjusting for all covariates, the following factors remained independently associated with OS: older age (HR = 1.03, 95% CI: 1.01–1.04, *p* < 0.05), N2 stage (HR = 1.71, 95% CI: 1.17–2.89, *p* < 0.05), N3 stage (HR = 1.86, 95% CI: 1.22–2.84, *p* < 0.05), MOM pattern (HR = 2.60, 95% CI: 1.96–3.46, *p* < 0.05), chemotherapy (HR = 0.63, 95% CI: 0.47–0.85, *p* < 0.05), and immunotherapy (HR = 0.54, 95% CI: 0.37–0.79, *p* < 0.05).

### 3.3 Kaplan–Meier survival analysis for the SOM group and the MOM group

**Figure 1:**
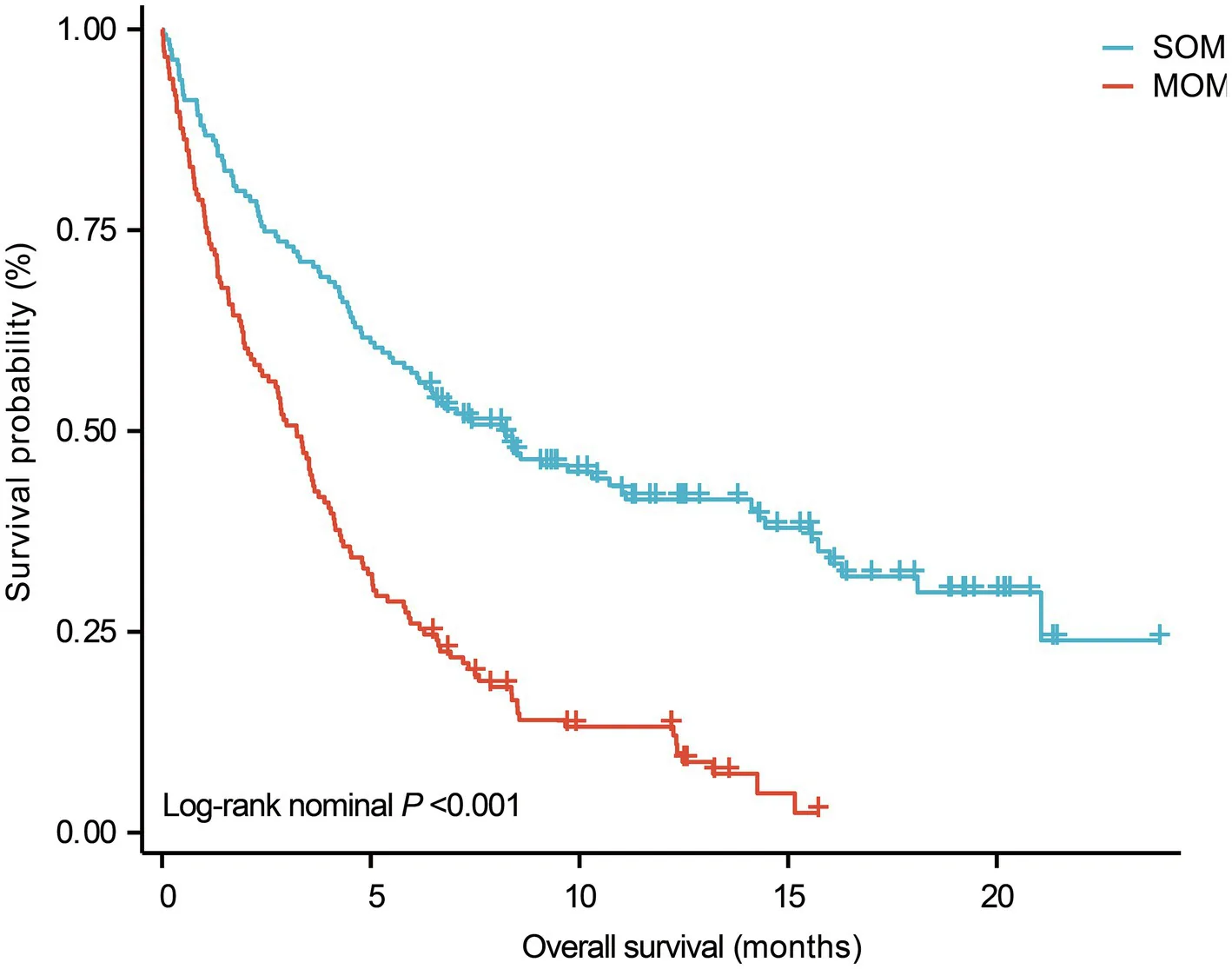
Kaplan–Meier survival curves of the SOM and MOM groups.

The Kaplan–Meier survival curves comparing OS between patients with SOM and MOM are shown in Figure 1. Patients in the MOM group exhibited consistently lower survival probabilities throughout the follow-up period than those in the SOM group. The median OS for the SOM group was 8.22 (95% CI: 5.66–10.78) months, whereas it was 3.22 (95% CI: 2.61–3.83) months for the MOM group. Additionally, the log-rank test showed a statistically significant difference between the two groups (log-rank *p* < 0.05).

### 3.4 Distribution of metastatic sites

**Figure 2:**
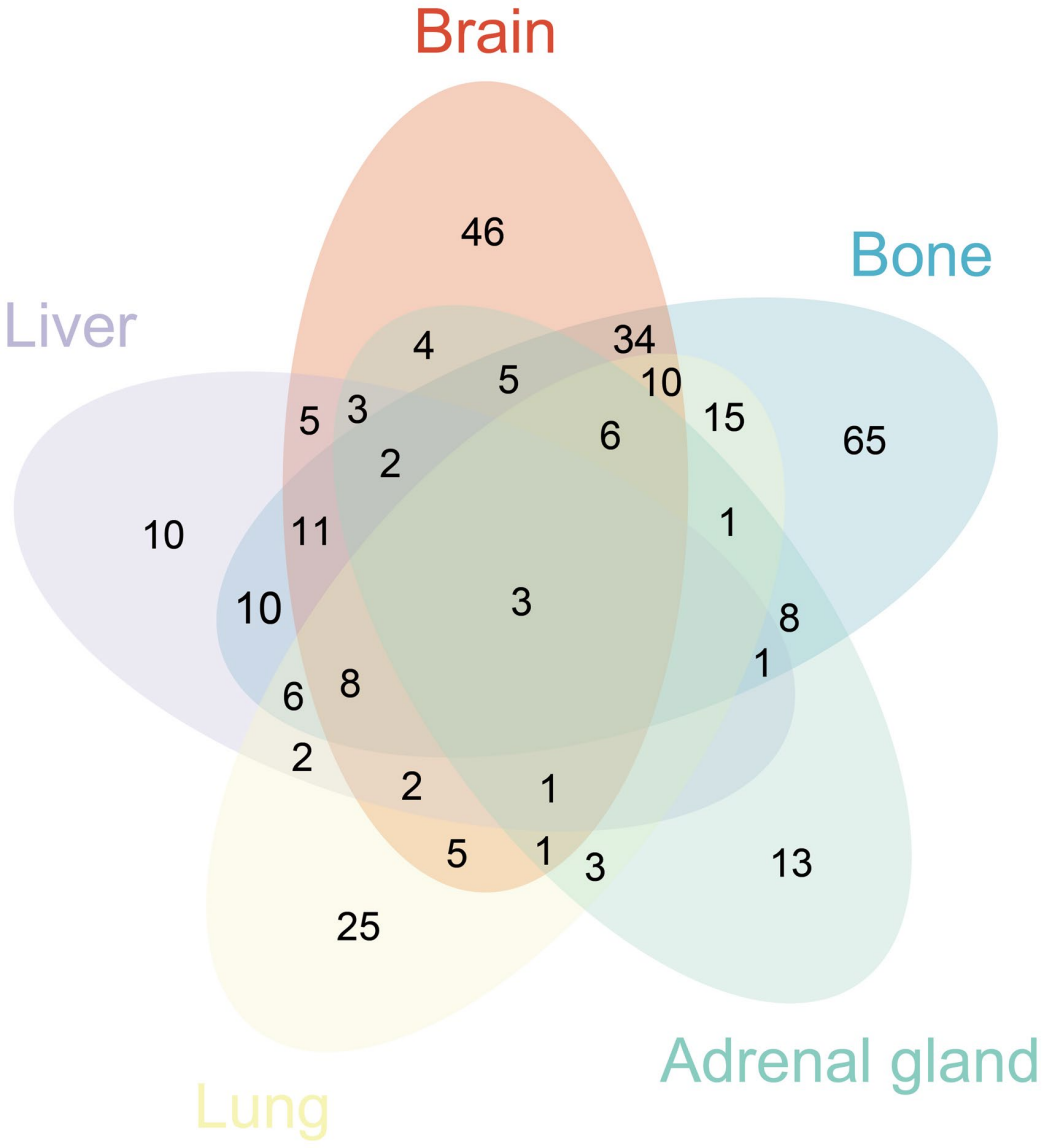
Venn diagram of metastatic organ distribution (*n* = 305).

The distribution of metastatic sites in the SOM and MOM groups is presented in Table 3 and Figure 2. In the SOM group, the most common site of metastasis was the bone (40.9%), followed by the brain (28.9%), lung (15.7%), adrenal gland (8.2%), and liver (6.3%). In the MOM group, bone metastasis remained the most frequent site (82.2%), followed by the brain (68.5%), lung (43.2%), liver (37.0%), and adrenal gland (26.0%).

**Table 3:** Distribution of metastatic sites in the SOM and MOM groups (*n* = 305).

Metastatic site	SOM (*n* = 159)	MOM (*n* = 146)
Bone, *n* (%)	65 (40.9)	120 (82.2)
Brain, *n* (%)	46 (28.9)	100 (68.5)
Liver, *n* (%)	10 (6.3)	54 (37.0)
Lung, *n* (%)	25 (15.7)	63 (43.2)
Adrenal gland, *n* (%)	13 (8.2)	38 (26.0)

### 3.5 Survival outcomes by specific metastatic site in the SOM group

**Figure 3:**
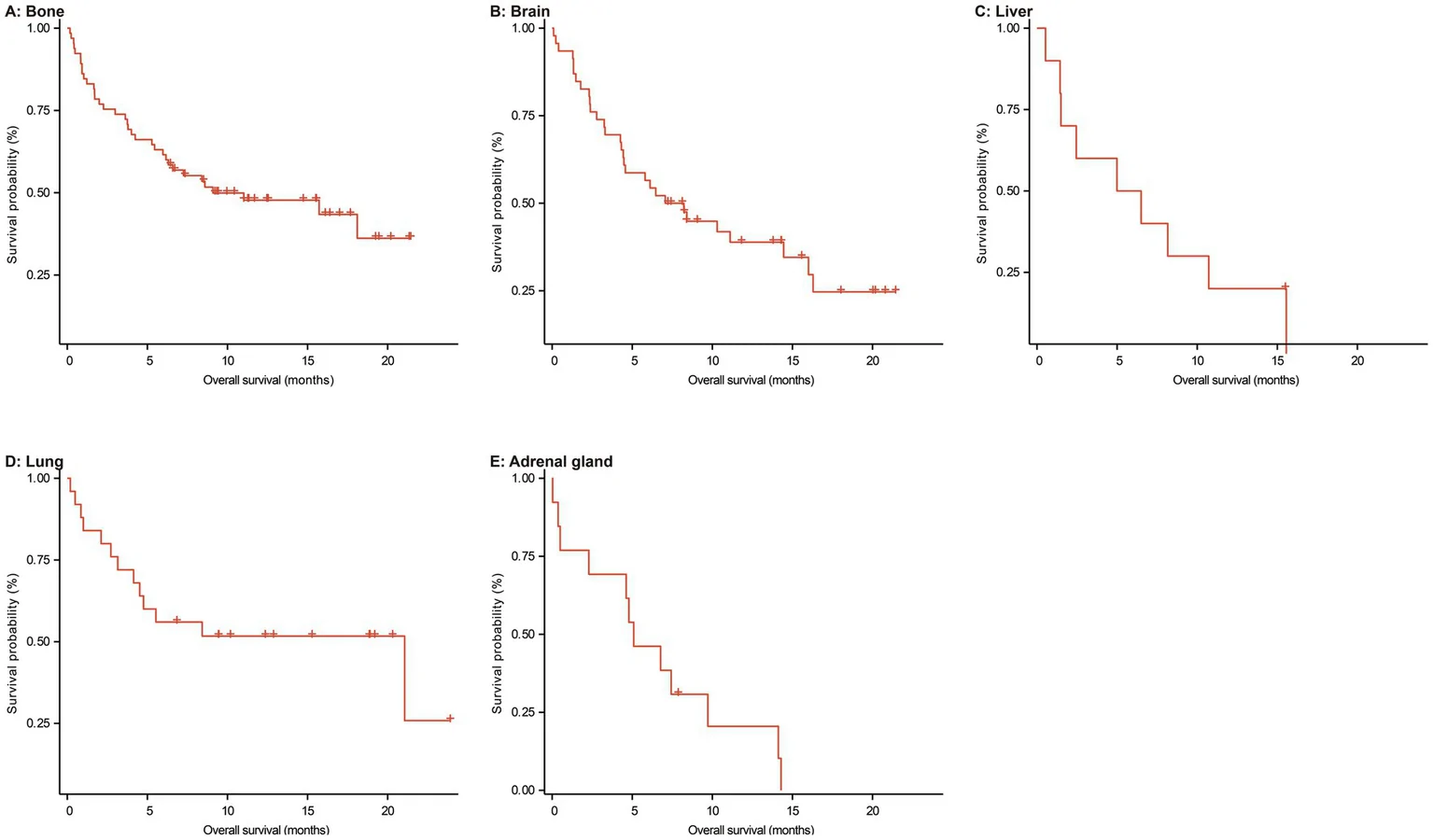
Kaplan–Meier survival curves of specific metastatic organs in the SOM group.

To further evaluate the prognostic impact of individual metastatic organs among patients with SOM, we performed the Kaplan–Meier survival analysis for each of the five metastatic sites: bone, brain, liver, lung, and adrenal gland. The survival curves are presented in Figure 3. The median OS for patients with isolated bone metastasis was 9.09 (95% CI: 0.22–17.96) months, for those with brain metastasis was 7.06 (95% CI: 3.83–10.29) months, for those with liver metastasis was 4.99 (95% CI: 0.00–11.27) months, for those with lung metastasis was 21.06 (95% CI: 1.93–40.19) months, and for those with drenal gland metastasis was 5.09 (95% CI: 2.57–7.62) months.

Pairwise comparisons using the log-rank test (detailed in Table 4) revealed that patients with adrenal gland metastasis had significantly worse OS than those with bone metastasis (*p* < 0.05) and lung metastasis (*p* < 0.05). No statistically significant differences were observed between adrenal gland metastasis and brain metastasis (*p* > 0.05) or between adrenal gland metastasis and liver metastasis (*p* > 0.05). Furthermore, there were no significant survival differences among the other comparisons: bone vs. brain (*p* > 0.05), bone vs. liver (*p* > 0.05), bone vs. lung (*p* > 0.05), brain vs. liver (*p* > 0.05), brain vs. lung (*p* > 0.05), and liver vs. lung (*p* > 0.05).

**Table 4:** Pairwise comparisons of OS among specific metastatic organ sites in the SOM group.

	Bone	Brain	Liver	Lung	Adrenal gland
Bone	1				
Brain	0.379	1			
Liver	0.053	0.188	1		
Lung	0.771	0.327	0.072	1	
Adrenal gland	**0.016**	0.069	0.505	**0.032**	1

## 4 Discussion

In this retrospective cohort study of 305 patients with metastatic NSCLC, we characterized the distribution patterns of metastatic involvement in five common organs (bone, brain, lung, adrenal gland, and liver) and evaluated their prognostic significance. Our results demonstrated that MOM was associated with significantly worse OS compared with SOM, with an adjusted HR of 2.60. Among patients with SOM, survival outcomes varied considerably across different metastatic sites, with lung metastasis showing the longest median OS of 21.06 months and liver metastasis showing the shortest OS of 4.99 months. Additionally, advanced N stage (N2 or N3), older age, and the lack of chemotherapy or immunotherapy were identified as independent adverse prognostic factors.

The finding that MOM confers a substantially poorer prognosis compared with SOM aligns with previous observations in the literature. A growing body of evidence has suggested that the number of involved metastatic organs is a robust predictor of survival in advanced NSCLC, often surpassing the predictive value of any single metastatic site (10, 11, 15). This phenomenon might be partly attributable to several interrelated factors. A higher metastatic burden generally indicates more aggressive tumor biology and greater genomic instability, which could lead to accelerated disease progression and resistance to therapy (16). Moreover, involvement of multiple organs often precludes effective local ablative therapies, such as stereotactic radiosurgery for brain metastases or radiotherapy for bone lesions, thereby limiting treatment options (17). In addition, it is plausible that the cumulative physiological impact of metastases across different organ systems can accelerate overall functional decline and reduce tolerance to systemic therapies (18). These considerations underscore the importance of stratifying patients not only by the presence or absence of metastasis but also by the extent of metastatic spread.

Regarding specific metastatic sites in the SOM subgroup, our study revealed a notable survival hierarchy. Patients with isolated lung metastasis had the most favorable prognosis, with a median OS exceeding 21 months, whereas those with isolated liver metastasis exhibited the poorest outcome at less than 5 months. Patients with bone, brain, or adrenal gland metastasis had intermediate survival between these extremes. The favorable outcome associated with lung metastasis could be related to the fact that intrapulmonary metastases, when confined to the lung, can sometimes be managed with local treatments such as radiation or surgery, and the preserved function of other vital organs allows for more aggressive systemic therapy (19). Conversely, liver metastasis has been increasingly recognized as a particularly adverse prognostic factor in NSCLC (20). One hypothetical explanation is that the liver is a central organ for drug metabolism, and the presence of metastatic lesions might alter the pharmacokinetics of chemotherapeutic agents and targeted therapies, potentially reducing their efficacy (21). Furthermore, the liver microenvironment has been suggested to promote immune tolerance, which may attenuate the response to immune checkpoint inhibitors (22). Together, these proposed biological features, while still speculative, highlight why liver metastasis may pose a formidable challenge in clinical management.

An unexpected observation in our study was the relatively short median OS (5.09 months) for isolated adrenal metastasis, which appears to contrast with several previous reports suggesting that adrenal metastases may confer a more favorable prognosis compared with other visceral sites (5, 23, 24). However, this comparison should be interpreted with great caution for several reasons. The sample size of patients with isolated adrenal metastasis in our study was relatively small (*n* = 13, representing 8.2% of the SOM group), resulting in wide confidence intervals around the median survival estimate (95% CI: 2.57–7.62 months), indicating substantial statistical uncertainty. Differences in patient populations, treatment patterns, and follow-up durations across studies could also account for the divergent results (25). Moreover, the pairwise comparisons were not adjusted for multiple testing, which increases the probability of a chance finding (type I error). It is, therefore, possible that the observed difference between adrenal and other sites may not be reproducible in larger cohorts. Furthermore, the adrenal gland may be a site of occult metastases that are detected later in the disease course, or adrenal involvement might coexist with other unmeasured adverse risk factors (26). Given the small subgroup size and the exploratory nature of these analyses, this finding should be interpreted as hypothesis-generating rather than confirmatory and requires validation in larger independent cohorts with appropriate adjustment for multiple comparisons.

Our multivariate analysis identified several other independent prognostic factors. Advanced N stage (N2 or N3) remained significantly associated with poorer OS after adjusting for metastatic pattern and treatment variables. This finding suggests that regional lymph node involvement, even in the presence of distant metastasis, contributes additional prognostic information beyond the extent of organ spread. N2 and N3 diseases may reflect a higher baseline tumor burden or a more invasive phenotype (27). The protective effects of chemotherapy and immunotherapy observed in our study are consistent with the current standard of care for advanced NSCLC (28, 29). Importantly, these associations persisted after controlling for metastatic pattern, indicating that the survival benefit of systemic therapies is not entirely explained by differences in disease distribution.

The major strength of this study is the head-to-head comparison of survival outcomes across all five metastatic sites within a strictly defined SOM cohort. This design eliminates confounding from multiple-organ involvement, enabling an unbiased assessment of site-specific prognostic effects. To the best of our knowledge, few previous studies have performed such systematic pairwise comparisons. The majority of previous reports either included mixed populations or lacked direct site-to-site analyses. By restricting our analysis to the SOM subgroup, we identified a prognostic hierarchy among isolated metastases that may inform clinical decision-making. In addition, the availability of detailed clinical data allowed adjustment for key confounders, including N stage, age, and treatment modalities.

Several limitations should be acknowledged. The retrospective, single-center design may introduce selection bias and limit generalizability. The sample size, while adequate for primary analyses, was relatively modest for subgroup comparisons, particularly for less common metastatic sites such as the adrenal gland and liver. Most notably, the small number of patients with isolated adrenal metastasis (*n* = 13) renders our finding regarding its poor prognosis preliminary and hypothesis-generating rather than confirmatory. The pairwise comparisons were not adjusted for multiple testing, increasing the risk of type I error. In addition, we did not stratify the SOM group by lesion number. Molecular profiling data (e.g., Epidermal Growth Factor Receptor (EGFR), Anaplastic Lymphoma Kinase (ALK), Ros Oncogene 1, Receptor Tyrosine Kinase (ROS1), Kirsten Rat Sarcoma Viral Oncogene Homolog (KRAS), B-Raf Proto-Oncogene, Serine/Threonine Kinase (BRAF), Mesenchymal-Epithelial Transition Factor (MET), Rearranged during Transfection Proto-Oncogene (RET), Programmed Death-Ligand 1 (PD-L1)) were unavailable, despite their well-established roles in survival and metastasis in advanced NSCLC. Future large-scale, multi-center studies are warranted to validate our findings in broader populations. Despite these limitations, our study provides clinically relevant insights into metastatic organ distribution and its prognostic value in advanced NSCLC. The systematic comparison across single-organ metastasis sites offers a reference frame for clinicians evaluating patients with limited metastatic spread. These findings support the routine assessment of both the number and location of involved metastatic organs in prognostic evaluation and treatment planning.

## 5 Conclusion

In this retrospective analysis of 305 patients with metastatic non-small cell lung cancer, MOM was independently associated with significantly worse OS than SOM (adjusted HR: 2.51). Among patients with SOM, survival varied considerably by the metastatic site, with lung metastasis conferring the longest survival and liver metastasis the shortest. Notably, isolated adrenal metastasis was associated with significantly worse OS than bone or lung metastases in pairwise comparisons, although this finding is based on a small subgroup and should be considered hypothesis-generating pending validation in larger cohorts. Advanced N stage (N2 or N3) and older age were identified as additional adverse prognostic factors, whereas chemotherapy and immunotherapy were associated with improved survival. These findings underscore the importance of considering both the extent and distribution of metastatic involvement in risk stratification for patients with advanced non-small cell lung cancer. The prognostic hierarchy identified among single-organ metastasis sites may help guide surveillance intensity and treatment prioritization, particularly for patients with limited metastatic burden. Future prospective studies incorporating molecular profiling and detailed treatment data are warranted to refine prognostic models and guide personalized therapeutic strategies.

## Data Availability

The original contributions presented in the study are included in the article/supplementary material, further inquiries can be directed to the corresponding author.
