# Correction: Metastatic organ distribution characteristics and prognosis in patients with non-small cell lung cancer

**DOI:** 10.3389/fmed.2026.2014576

**Published:** 2026-10-13

**Authors:** Jianlei Zhu, Wenyan Pan, Yanyang Wang

**Affiliations:** 1 Department of Radiotherapy, Ningxia Medical University General Hospital, Yinchuan, Ningxia, China; 2 Department of Medical Oncology, Yinchuan First People’s Hospital, Yinchuan, Ningxia, China

**Keywords:** metastasis, non-small cell lung cancer, organ distribution, overall survival, prognosis

Affiliation “Department of Radiotherapy, Ningxia Medical University General Hospital, Yinchuan, Ningxia, China” was erroneously omitted for author “Wenyan Pan”. The conflict of interest statement has been corrected to ‘The author(s) declared that this work was conducted in the absence of any commercial or financial relationships that could be construed as a potential conflict of interest’.

Affiliation “Department of Radiotherapy, Ningxia Medical University General Hospital, Yinchuan, Ningxia, China” was omitted from the paper. This affiliation has now been added for author “Wenyan Pan”.

Affiliation “Department of Medical Oncology, Yinchuan First People's Hospital, Yinchuan, Ningxia, China” was erroneously included in the paper. This affiliation has now been removed for author “Wenyan Pan”.

The original version of this article has been updated.

